# What oncologists tell patients about survival benefits of palliative chemotherapy and implications for informed consent: qualitative study

**DOI:** 10.1136/bmj.a752

**Published:** 2008-07-31

**Authors:** Suzanne Audrey, Julian Abel, Jane M Blazeby, Stephen Falk, Rona Campbell

**Affiliations:** 1 University of Bristol, Department of Social Medicine, Bristol BS8 2PR; 2 Weston Area Healthcare Trust, Uphill, Weston-super-Mare, BS23 4TQ; 3 University of Bristol, Clinical Sciences at South Bristol and Department of Social Medicine, Bristol BS8 2PR; 4 Divison of Surgery, Head and Neck, University Hospitals Bristol NHS Foundation Trust, Bristol BS2 8HW; 5 Bristol Haematology and Oncology Centre, University Hospitals Bristol NHS Foundation Trust, Bristol BS2 8ED

## Abstract

**Objective** To examine how much oncologists tell patients about the survival benefit of palliative chemotherapy during consultations at which decisions about treatment are made.

**Design** Qualitative study in which consultations were observed and digitally recorded.

**Setting** Teaching hospital and district general hospital in south west England.

**Participants** 37 patients with advanced non-small cell lung cancer (n=12), pancreatic cancer (n=13), and colorectal cancer (n=12); and nine oncologists, including four consultants and five registrars.

**Main outcome measures** All recordings were transcribed completely, anonymised, and electronically coded with ATLAS.ti. Constant comparison was used to identify themes and patterns. The framework method of data management, in which data were charted, was used to aid transparency of interpretation.

**Results** During the consultations, information given to patients about survival benefit included numerical data (“about four weeks”), an idea of timescales (“a few months extra”), vague references (“buy you some time”), or no mention at all. In most consultations (26/37) discussion of survival benefit was vague or non-existent.

**Conclusions** Most patients were not given clear information about the survival gain of palliative chemotherapy. To aid decision making and informed consent, we recommend that oncologists sensitively describe the benefits and limitations of this treatment, including survival gain.

## Introduction

Every year in the United Kingdom many thousands of patients are told they have incurable cancer and are offered palliative chemotherapy. Such treatment commonly requires frequent visits to hospital and considerable interruption to everyday activities. Because of the considerable toxicity and the modest survival benefits, decisions about treatment can be extremely difficult.

*The NHS cancer plan *has emphasised the importance of good communication between patients and the staff caring for them.^1^ Many patients want more information about their disease and treatment options,^2^ ^3^ and this is important if patients are to exercise informed consent. The Department of Health guide *Consent—what you have the right to expect* indicates that “patients must be given enough information to make a decision” and suggests the sort of information health professionals should offer, including “the benefits they hope will result” and “the chances of getting such benefits.”^4^ There is an increasing body of literature concerned with communication and decision making,^5^ ^6^ ^7^ ^8^ and it is recognised that doctors benefit from training in “breaking bad news.”^9^ ^10^ For patients with advanced cancer, however, there is wide variation in the amount of information given, and decision making aids are scarce.^11^ ^12^ ^13^ ^14^

### Survival benefit

Survival benefit is often the primary outcome measure in clinical research relating to palliative chemotherapy. This suggests a difference in perspective between palliative care clinicians, for whom “palliative” refers to improvement in quality of life, and those clinicians who focus on prolonging life.^15^ ^16^ Emphasising survival gain, however, might not be in conflict with the wishes of patients. Many patients with advanced cancer prioritise survival over quality of life^14^ and oncologists might “collude” with them in attempting to do something active about the disease.^17^ The offer of active treatment with palliative effects can support patients through a process of adjustment from the distress of diagnosis towards goals other than long term survival.^18^ But if survival benefit is not discussed when treatment decisions are being made, there can be a considerable gap between patients’ hopes and what can usually be achieved.

The search for effective treatments continues but, at the advanced stages of cancer, survival gain from palliative chemotherapy tends to be months rather than years. Statistics relating to survival benefit can be contested, prompting concerns among clinicians about how patients can make informed decisions if experts do not agree among themselves.^19^ Furthermore, there are concerns that the “intrusiveness of unfavourable numbers” can undermine healthcare relationships and destroy hope.^20^ The challenge for oncologists, and multi-disciplinary cancer teams, is to communicate sufficient information to enable patients to make informed decisions based on realistic aspirations^21^ but to do so in a sensitive manner and at the patient’s pace.^22^

We focused on qualitative data from oncology consultations during a study of patients’ experiences of treatments (ASPECTS) and examined the extent to which survival gain was discussed when patients were offered palliative chemotherapy.

## Methods

### Study design and setting

Data for this paper come from the larger ASPECTS study, which was conducted in the south west of England. The study used qualitative research methods to describe patients’ experiences of palliative chemotherapy and to explore how the decision making process might be improved in the light of those experiences. During the main study an experienced qualitative researcher interviewed patients before they saw the oncologist; recorded and observed the initial oncology consultation at which palliative chemotherapy was discussed; and interviewed patients again, at least once, in the weeks that followed. Relevant oncologists consented to the observation and recording of consultations and were also interviewed towards the end of the study. Fieldwork took place in patients’ homes, a large teaching hospital, and a district general hospital.

Three common cancers were chosen: colorectal, non-small cell lung, and pancreatic. Trials are ongoing but current UK guidelines suggest that palliative chemotherapy might extend median survival by up to two months for non-small cell lung cancer^23^ and from three to four months to five to six months for pancreatic cancer.^24^ For colorectal cancer median survival can increase from five to nine months to between 7.5 and 14 months with monotherapy and by a further three to five months with combination therapy.^25^ ^26^

### Recruitment

For the main study, clinical members of the research team identified patients according to cancer site at relevant multi-disciplinary team meetings. Patients were considered suitable for the study if they had locally advanced or metastatic disease, had been given a diagnosis, and had been offered an appointment to see an oncologist. Relevant patients were informed about the study and asked if they would be willing to participate. Those who expressed an interest were given an information leaflet and contacted by the qualitative researcher. At a subsequent meeting, the researcher explained the study again and patients who agreed to participate signed the consent form. At each stage it was made clear to patients that their medical care would be unaffected whether or not they took part.

Forty five patients with advanced cancer were recruited to the main study, 15 with each type of cancer. This represents half of those who were identified as suitable for the study (45/90). The main reasons for non-recruitment were administrative difficulties in contacting the patient because of the brief time period before their first appointment with the oncologist (n=16); patients refused with no clear reason given (n=11); patients or spouses indicated the patient was too unwell or anxious to be interviewed (n=9); or patients were unsuitable for another reason—for example, elderly patients with dementia (n=6).

When appropriate we provided an information leaflet, letter of invitation, and consent form for partners and carers. Thirty three partners or carers were recruited, indicating the extent to which this experience was shared by patients and those closest to them.

### Process

The consultations were digitally recorded, and a non-participant observed the consultation to capture non-verbal communication. Video recording was not practicable in the context of busy oncology clinics, where the room allocated for a specific patient was not always known in advance. In addition, the presence of the researcher, as a non-participating observer, whom the patients had already met and would meet again was thought to be more appropriate and was in fact regarded by some patients and their carers as a “friendly face” at this difficult time. The researcher did not take notes during consultations as this was likely to be distracting for participants and might have prompted concerns about what was being noted and why. The researcher sat quietly in the corner of the room and completed a reflective diary as soon as possible after the consultation. All the recordings were fully transcribed and anonymised to protect confidentiality, and field notes were inserted in the text of transcripts to highlight contextual issues where appropriate.

### Analysis

During analysis we used the method of constant comparison derived from grounded theory.^27^ ^28^ We read and re-read the transcripts to become familiar with the data and revisited them at pertinent points throughout the analysis. Data were electronically coded with ATLAS.ti.^29^ Codes and themes were derived from issues raised by participants, experiences that recurred in the data, and the research aims. To aid analysis, we adapted and used the Framework method of qualitative data management.^28^ ^30^ This entailed “charting” the coded data. To explore information giving during the consultations, we developed a chart (table 1). Practitioners of Framework often handle large amounts of data and, to make them more manageable, summarise the original data before entering them onto charts. The team was concerned to capture this stage of interpretation and so we entered original data relevant to the purpose of treatment and survival benefit onto a large primary chart that was read across rows for each patient and down columns to compare experiences. During this process, variations in the discussion of survival gain emerged.

**Table 1 tbl1:** Example of Framework charting for data management in main study

Patient ID	Sex	Age (years)	Cancer site	Clinician ID	Treatment decision	Quotes from oncologist	
						Purpose of treatment	Survival benefit discussion
301	M	65	Lung	101	Chemotherapy offered and accepted	It’s a relatively uncommon situation and normally if you can’t do an operation then there’s very little chance of curing a lung cancer I’m afraid (cont…)	If we can’t achieve that, a cure, then we’re looking to control it, shrink it, get rid of the cough, buy time. In this situation I think it’s a bit more unclear (cont…)
302	F	57	Pancreas	102	Chemotherapy offered and accepted	Now none of the drugs that have been developed are cures and they don’t work for everybody. About 30% of people who receive them (cont …)	You’ve got to be fit for it. Now with chemotherapy, the benefit is not seen at all in the people who would do very badly anyway, right (cont…)
303	F	69	Colorectal	103	Chemotherapy offered and accepted	Now the situation is that chemotherapy has its pros and its cons. The problem is that when the cancer is widespread like this, treatment is what we call palliative (cont…)	The aim of chemotherapy therefore is to keep you going, not just to give you extra quantity of life but extra quality of life. The evidence is that people who respond to treatment, they get both (cont…)

We removed data less pertinent to this central issue to produce smaller charts with a focus on survival gain. During this phase we eventually categorised the survival benefit information as “numerical,” “idea of timescales,” “vague,” and “not discussed.” These charts were sorted—for example, by patients’ sex and cancer site—to compare data relating to these subgroups and look for patterns. Further reading and analysis, including revisiting the primary chart and transcripts, identified triggers and barriers to discussion of survival gain. This method of data management promoted transparency, enabling the research team to collectively scrutinise each stage.

### Participants

We observed and digitally recorded 37 oncology consultations (three patients were too ill to keep the appointment with the oncologist; two consultations were not recorded; one patient refused to see an oncologist; and the possibility of cure, judged to be beyond the definition of survival benefit for this paper, was mentioned during two consultations). We have not included data from interviews before and after the consultation because our primary concern was with information given to the patient by the oncologist at this initial consultation. We wanted to assess whether the information provided at this stage was “enough information to make a decision” and whether it included “the benefits they [the oncologists] hope will result” and “the chances of getting such benefits” as the Department of Health guidance on seeking informed consent to treatment suggests that it should.^4^ Table 2 describes the patients included.[Table tbl2] The nine oncologists who saw these patients were mixed in terms of age, experience, and sex; and included four consultants and five registrars. (Further details are not given to protect confidentiality.)

**Table 2 tbl2:** Details of 37 patients whose oncology consultation was studied

	No of patients
Men	13
Women	24
Age (years):	
≤50	1
51-59	8
60-69	11
70-79	16
≥80	1
Cancer site:	
Colorectal	12
Lung	12
Pancreas	13

## Results

### Purpose of treatment

Towards the beginning of the consultation all the patients were informed that their cancer could not be cured. For example:

Oncologist 102: None of the drugs that have been developed are cures, and they don’t work for everybody.

Oncologist 104: Whatever we use, it won’t get rid of it.

Oncologist 112: We know that we’re not going to be able to cure your cancer for you.

Having established that the aim was not cure, the oncologists explained the main purpose of chemotherapy either in terms of shrinking, slowing down, controlling, or stabilising the tumour; improving symptoms such as pain and weight loss; and/or improving quality of life—for example, enabling patients to feel “as well as possible for as long as possible.”

Patients who were offered chemotherapy were given the names of relevant drugs; information about the treatment regimen; and details of common side effects, such as nausea, and measures to alleviate them. Patients were also warned of the increased risk of life threatening infections and what to do in an emergency. Those who accepted chemotherapy signed consent forms to enable the oncologists to order drug treatment. Patients were told that this did not commit them to having the treatment if they subsequently decided against it, and that the nurses responsible for administering chemotherapy would explain the treatment again and answer further questions. While this was described as a staged consent process, none of the patients who consented at the initial oncology consultation subsequently refused treatment.

### Survival benefit

Although there was consistency in informing patients that a cure was not being sought, the amount of information given about survival benefit varied considerably (table 3). This ranged from giving numerical data, such as “about four weeks”; through an idea of timescales, such as “a few months extra”; to vague references, including “buy you some time”; to not being mentioned at all. During the recorded consultations, only six of the 37 patients were given numerical data about the survival benefit of treatment. These included three of the 23 patients who accepted palliative chemotherapy. In most consultations (26/37) the discussion of survival benefit was either vague or non-existent.

**Table 3 tbl3:** Treatment decisions and discussion of survival benefit in oncology consultations

Information about survival benefit	Total	Treatment decision			
		Chemotherapy offered and accepted	Chemotherapy offered and refused	Chemotherapy not offered	Further appointment
Numerical data	6	3	2	1	0
Idea of timescales	5	5	0	0	0
Vague	18	14	1	2	1
Not discussed	8	1	2	3	2
Total	37	23	5	6	3

In qualitative research with small numbers we are not looking for statistical significance but we did scrutinise the data to see if there were any obvious patterns. None was found in relation to the sex or age of the patient, hospital site, cancer site, treatment decision, or the actual survival of the patient (22 patients died during the course of the fieldwork). Individual oncologists did not adopt a consistent approach with all patients in relation to the amount of information given about survival benefit (table 4). Registrars seemed less likely to discuss the issue, but the numbers are too small to draw any firm conclusions.

**Table 4 tbl4:** Information on survival benefit given in oncology consultations

Oncologist	Numerical data	Idea of timescales	Vague	Not discussed	Total
101	—	5	3	—	8
102	2	—	3	—	5
103	1	—	5	3	9
104	3	—	2	1	6
107, 108, 110, 112, 113	—	—	5	4	9
Total	6	5	18	8	37

### Triggers and barriers

By looking at the qualitative data in more detail we could discern some triggers and barriers to discussion of survival benefit. These are examined below and illustrated with extracts from the transcripts.

#### Triggers

A few patients, or their relatives, specifically asked for details. For example:

Son: And what’s the best you would expect with that?

Oncologist 104: It may improve it by two to three months.

Patient 335: Mm.

Oncologist 104: [to wife who was distressed] Is that what you thought?

Wife: No, I’m afraid I didn’t give it much thought, not in actual months.

Son: I’m sorry I had to ask that mum, because it’s an important part of making the decision isn’t it?

Wife: Yes, of course. I know that, yes.

Son: If you’re going to go through lots of pain and problems.

Patient 335: Oh yes. Em, I em, I would have asked it anyway if my son hadn’t.

Chemotherapy was offered and accepted; the patient died two months later.

Sometimes the oncologist volunteered the information to give a realistic expectation of what the treatment could achieve:

Oncologist 101: Now if somebody has chemotherapy we’re, we’re not unfortunately talking about people living years longer, we’re talking about months on the whole. Some lucky people may live some years longer, but that’s not the average expectation.

Chemotherapy was offered and accepted; patient 309 died five months later.

Discussion of survival benefit seemed to be helpful for some patients who decided that they did not want chemotherapy and enabled them to justify their decision, especially to family members who wanted them to “fight” the disease:

Patient 315: Off the record, do you think there’s any benefit for me to have treatment?

Oncologist 103: The problem is I only know that after the event.

Patient 315: After the event, yes.

Oncologist 103: My problem is I see it both sides.

Patient 315: Yeah.

Oncologist 103: I’ve got some patients who’ve done very well with these treatments and have lived for much longer than frankly I would have expected.

Patient 315: Hmm mm.

Oncologist 103: I have other patients who either have a lot of side effects with the treatment and no benefit, or clearly go through it all and shortly after it’s playing up, and what would be really helpful isif I could tell you which of those two folk there you were going to be and the whole problem in this situation is that I can’t.

Patient 315: My worse nightmare would probably be to have some treatment and end up back in hospital with another ailment.

Later in the consultation:

Patient’s wife: You’re going to fight it. You said you would.

Patient 315: Yeah but it doesn’t mean to say it’s only going to be nine months I mean it might be 12, it might be 15, it might be.

Oncologist 103: Averages are dangerous statistics.

Patient 315: Yeah, you never know. I said to you before I’d sooner have a short amount of time with a bit of bonus to it, a bit of benefit. If I had to go into hospital for five weeks every day and, and not benefit from it and even catch something worse and end up back in hospital for the rest of me life basically, then I’d have to top myself.

Chemotherapy was offered and refused; the patient died three months later.

Discussion of survival benefit was also helpful for clinicians who had judged that patients were too ill to tolerate the treatment. In such cases, oncologists could point to the statistics on low survival benefit to show that they were not withholding valuable treatment:

Oncologist 102: And even if it does work, it can prolong life, but only by about four weeks. So it’s not the answer, we know that, but it can be a dangerous thing, and shouldn’t just be thrown about.

Chemotherapy was not prescribed; patient died two weeks later.

#### Barriers

Some of these apparent triggers could also be barriers to the discussion of survival benefit. If patients made it clear from the outset that they did not want chemotherapy, then the treatment might not be discussed in any detail:

Patient 327: Chemotherapy that is completely out. I don’t want that at all.

Oncologist 104: OK, right.

Patient 327: If I’m offered the opportunity of radio . . .

Oncologist 104: Yeah.

Patient 327: Em, and that would ease the pain . . .

Oncologist 104: Yeah.

Patient 327: I’m quite happy to have that.

Patient refused chemotherapy and received radiotherapy; died three months later.

If oncologists judged patients to be too unwell for chemotherapy, the conversation might be steered away from the survival benefit of chemotherapy towards recommending other medication:

Oncologist 103: I don’t think your general condition now would tolerate chemotherapy quite honestly.

Patient 334: Well no, I thought it might buy me some time, but I mean . . .

Oncologist 103: And I think the problem is that because you’ve become so weak with it and lost so much weight. . .

Patient 334: And you don’t want to eat.

Oncologist 103: Absolutely, and that’s one of the commonest symptoms that the get-up-and-go gets up and goes, and one just doesn’t want to.

Patient 334: Yes, and my get-up-and-go’s gone.

Oncologist 103: Have you tried steroids or anything like that?

Patient 334: No, I, no.

Oncologist 103: Right. Well I think that will be a worthwhile thing to do, is for you to have some steroids and something to stop them upsetting your stomach.

Steroids were prescribed; the patient died six weeks later.

Emphasising the other benefits of chemotherapy could also divert the conversation away from survival benefit. This patient asked about life expectancy and survival benefit, saying he was quite happy to be given figures:

Oncologist 112: OK. So we, in some people, in some cases it can actually really help with their cancers but in a lot of patients it can help with the symptoms.

Wife: Yes.

Oncologist 112: And this is why we give it.

Patient 339: Right

The oncologist did not give figures for life expectancy or survival benefit. Chemotherapy was offered and accepted; the patient was alive six months after consultation.

Patients and their partners sometimes blocked the discussion:

Oncologist 103: Do you want me to tell you what the statistics are?

Husband: Not particularly, do you?

Patient 303: No.

Husband: No.

Oncologist 103: The problem with statistics is they don’t tell you which, which side, and it comes down to, Is it worth it, going through this treatment? Is there something that’s worthwhile going through all this treatment, that I want to live longer or. . .

Husband: I think we have to make that decision as well. I don’t think we want to go into the statistics.

Patient 303: Yeah.

Chemotherapy was offered and accepted; the patient was alive 18 months after consultation.

Extracts from a consultation with a patient who was distressed at the way in which she had previously been given her diagnosis, however, show the possibility of imparting information about survival benefit while respecting a patient’s wish not to discuss the prognosis (box 1). The oncologist acknowledged the difficulties of being precise about survival gain but did indicate that the “average” was “not great.” The inclusion of information about range, as well as median survival gain, softened the message and the patient seemed satisfied with the level of information she had been given.

**Box 1 Giving information about survival gain to a patient without discussing prognosis**

Oncologist 101: In terms of benefit we’d need to know also how much extra benefit in terms of survival that people will get. The average is not very great. It’s a few months. But the problem is that some people will get, you know, a year or two. Some people get no benefit at all. And that’s why we can only sort of see whether it’s a useful treatment by trying it out for one or two treatments to see, you know, if you’re somebody who gets a good response to the chemotherapy. If we do that and we find that you are responding well, the tumour’s shrinking and everything’s getting better then we will go on up to four or a maximum of six treatments in total. And we’d also ask the question “Is there any benefit from radiotherapy at the end of that period of treatment?” If you weren’t getting benefit from the chemotherapy and you were still short of breath then we would be saying well perhaps we should, could consider some radiotherapy and stop the chemotherapy. So we’ll be making those decisions as we go along.
Patient 346: As we, yeah. . .
Oncologist 101: And talking it through with you as we go step by step. That’s a lot of information all at once, I’m sorry about that.
Patient 346: It’s all right, that, that’s why I have my, my daughter here.

Chemotherapy was offered and accepted; the patient was alive three months after consultation.

## Discussion

For patients with advanced cancer to make informed decisions about palliative chemotherapy, oncologists need to describe the benefits and limitations of this treatment, including survival benefit. The oncology consultations studied here were important for several reasons. These were the first consultations with an oncologist after patients had been told their cancer was advanced and a cure was not being sought; at which patients expected to receive information from the oncologist about further treatment options; and when most patients consented to receive palliative chemotherapy.

### Impact on decision making

Study of the triggers and barriers to a discussion of survival benefit during the consultations showed clear implications for informed consent. In some oncology consultations the decision making process included giving patients information about the limited survival benefit of treatment (box 2). For example, a direct question was asked and answered giving at least an idea of timescales; the patient refused treatment but was aware of potential benefits including survival benefit; the patient was informed of the reason why active treatment was not being offered; and the oncologist volunteered information to encourage realistic expectations of what could be achieved.

**Box 2 Triggers and barriers to informed consent in oncology consultations**

**Triggers: survival benefit discussed**

**Patient**

- Asking direct question*
- Justifying refusal

**Oncologist**

- Responding (numerical data/idea of timescales)
- Justifying no active treatment
- Volunteering information (realistic expectations)

**Barriers: survival benefit not discussed or information is vague**

**Patient**

- Is patient assuming lengthy survival?
- Not wanting treatment
- Blocking*

**Oncologist**

- Focusing on other benefits (symptom relief)
- Is patient aware of potential benefits?
- Responsibility to (sensitively) inform

*Or by partner/carer, with patient’s agreement.

We identified barriers to the discussion of survival benefit that might undermine informed consent. If the oncologist focuses on the benefits of palliative chemotherapy in terms of control of symptoms and quality of life, but omits information about survival benefit, the patient might assume much greater potential to prolong life than is likely to be the case. Conversely, when patients decline the offer of palliative chemotherapy without a discussion of the potential benefits, including survival gain, they might be basing their decision on incomplete or inaccurate information. Perhaps most difficult of all is when a patient, or their partner or carer, makes it clear that they do not want to receive any more bad news. Talking about life expectancy can seem cruel at this point. But, as has been shown, supplying basic information about the survival benefit of treatment need not entail giving “intrusive” data about prognosis (see box 1).

### Do patients want to know?

During the ASPECTS study, although patients and their partners sometimes indicated that they did not want to discuss prognosis at this stage, there was no clear evidence that they did not want information about survival gain, and so it cannot be argued that clinicians were simply responding to patients’ preferences. Rather it seems that there is a tendency among oncologists to conflate the two concepts, at least in terms of what they consider might be helpful to the patient. At oral presentations of these data (Joint meeting of the Society for Social Medicine and the International Epidemiological Association (European Federation), University of Cork, Ireland, September 2007, and Annual research conference of Avon, Somerset, and Wiltshire Cancer Research Network, May 2008), some clinicians have suggested survival gain can be discussed only in relation to overall life expectancy, arguing, for example, that an additional week can be very important to someone who might have only a few weeks to live but can seem less important to someone who might be expected to live several more years. While this might be the case, patients are unable to make this assessment if they are not given the relevant information. Furthermore, it could be argued that patients might accept chemotherapy if they can look forward to many months or years of life after the treatment, but prefer their final weeks to be free from the potential side effects. Such arguments can only be speculative if the subject of survival gain is not broached during the decision making process. In practice, giving information about survival gain might prompt patients to ask about prognosis or, as has been illustrated here in relation to patient 346, patients might indicate that they do not want to discuss prognosis at this stage.

Most patients have an ongoing relationship with their doctor and not all information is given at one consultation. For patients with advanced cancer, however, it would be inappropriate to suggest that information about available treatments, which could influence their decision making, can be given after the treatment has started.

Giving comprehensible and appropriate information about survival benefit is extremely difficult. In addition, the reluctance to inform patients of the limited survival gain of palliative chemotherapy might be motivated by a desire to “protect” patients from bad news, as was once the case in relation to giving patients a diagnosis of cancer. The reluctance to address these difficulties and sensitivities, however, might be hampering patients’ ability to make informed decisions about their future treatment.

### Strengths and limitations

There have been few sound qualitative studies exploring patients’ experiences of palliative chemotherapy. These data contribute to this important yet under-researched topic. As with all qualitative studies, we did not have a large number of participants. Yet the range of patients and oncologists involved, and the inclusion of three cancer sites and two hospitals, suggest that the findings could be transferable to other settings. A further strength is the examination of data from consultations as they occurred, rather than retrospective interviews with patients or their oncologists.

The sample of patients comprised a higher proportion of men than might be expected among the broader population of patients with these cancers. The ratio of 24:13, however, is in line with the wider ASPECTS sample and with those who were eligible but not recruited to the study. This would suggest that the recruitment process was not biased in relation to sex. Furthermore, there was no evidence that sex affected the discussion of survival benefit.

Our results confirm previous research suggesting that there is wide variation in the amount of information given to patients.^11^ ^12^ ^13^ ^14^ As with all research, it is not possible to rule out Hawthorne effects.^31^ ^32^ The presence of a researcher who observed and recorded the consultations might have changed the content of discussions. It could be argued, however, that this might have encouraged oncologists to be more thorough and to provide patients with more, rather than less, information.

### Implications for practice

It is now widely accepted that patients should be told if they have cancer^33^ and current guidance for oncologists in “breaking bad news” includes how to convey an unwelcome diagnosis. Accordingly, during ASPECTS, the oncologists reinforced the diagnosis of advanced cancer and explained that, in prescribing chemotherapy at this stage, a cure was not being sought. But current guidance also places emphasis on informed decision making. Evidence from ASPECTS suggests that oncologists attempt to meet this obligation by giving details of the potential side effects of chemotherapy with much less time given to discussing the possible benefits of treatment. Nevertheless, most patients accepted chemotherapy. This seems to be in line with the argument that patients will risk negative impacts on quality of life for survival gain.^14^ It is therefore important that patients with advanced cancer are made aware of the limitations of that survival gain during the decision making process. While it seems unlikely that this will change the treatment decision for many patients, it will contribute to narrowing the gap between what oncologists can currently offer and what some patients hope for.

The priority of informing patients has to be balanced against the emotional and psychological welfare of those who are distressed by a diagnosis of incurable cancer. Rather than evading this difficult subject, we recommend that oncologists receive support and training in how to communicate relevant information about survival benefit to their patients. Median survival gain is a difficult concept and can be open to misinterpretation but simple reassuring messages that could be given might include: “We are going to do our best for you”; “Our aim is to relieve your symptoms and improve your quality of life”; “We also need to consider what you would gain from this treatment in terms of extra time”; “The average gain may be a few months but very few people are “average” and so some people get quite a bit more than that, and sadly some people get no benefit at all.”

Patients’ understanding of survival gain is also pertinent to the debate about access to drugs through the National Health Service. While data about survival gain are included in National Institute for Health and Clinical Excellence (NICE) guidance for healthcare professionals, they are omitted from the “information for the public.”^34^ ^35^ ^36^ ^37^ ^38^ Though the intent might be to reduce distress, this can reinforce the gap between patients’ hopes and what can usually be achieved. It might also heighten concerns that valuable lifesaving treatments are being withheld for purely economic reasons. Consideration should be given as to whether, and how, NICE should communicate this important information to the public.

Greater awareness of survival gain might enable some patients to adjust their aspirations. Others will undoubtedly pin their hopes on being the exceptional patient who flouts the available evidence. It is not the job of the oncologist to deny such hope, and few would want to do so. The oncologists in this study acknowledged that current palliative chemotherapy regimens are not the answer to the treatment of advanced cancer. Along with their patients, they hope for something better in the future. In the meantime, by sensitively narrowing the gap between unrealistic expectations and current evidence, oncologists can be closer to fulfilling their responsibility of assisting patients to make informed decisions.

**What is already known on this topic**

Survival benefit is often a primary outcome measure in clinical research in palliative chemotherapy and is an important concern to patients
The survival benefits of palliative chemotherapy are modest
Current UK health policy places emphasis on the importance of informed consent

**What this study adds**

Most patients are not given clear information about the survival benefit of palliative chemotherapy, with consequent implications for decision making and informed consent
Training for oncologists should include guidance on how to inform patients about the survival benefit of palliative chemotherapy
Consideration should be given as to whether, and how, NICE should include this important information in their guidance leaflets for the public

## Notes

We thank all the patients and their families who shared their experiences with us. Their contribution to the research is invaluable. We are also grateful to the oncologists who were willing to be observed and recorded. Polly Nugent drafted an initial literature review. Jen Jamieson transcribed the recordings and was a source of considerable support during the fieldwork.

Contributors: JA had the initial idea for the ASPECTS study. RC, JA, and JMB designed the study and wrote the successful research proposal. JA and RC undertook pilot interviews. JA and JMB helped with patient recruitment. SA undertook the fieldwork for the main study and coded, charted, and analysed the data for this paper, which was scrutinised and discussed by the other authors. SA wrote the first draft. All authors commented on and contributed to the final draft. RC is guarantor.

Ethical approval: North Somerset research ethics committee, ref 05/Q2003/46.

Provenance and peer review: Not commissioned; externally peer reviewed.

## Notes

**Cite this as:** *BMJ* 2008;337:a752
